# Chimeric mitochondrial peptides from contiguous regular and swinger RNA

**DOI:** 10.1016/j.csbj.2016.06.005

**Published:** 2016-06-29

**Authors:** Hervé Seligmann

**Affiliations:** Unité de Recherche sur les Maladies Infectieuses et Tropicales Émergentes, Faculté de Médecine, URMITE CNRS-IRD 198 UMER 6236, Université de la Méditerranée, Marseille, France

**Keywords:** RNA–DNA differences, Bijective transformation, Nucleotide substitution, Proteome, Systematic deletions, delRNA

## Abstract

Previous mass spectrometry analyses described human mitochondrial peptides entirely translated from swinger RNAs, RNAs where polymerization systematically exchanged nucleotides. Exchanges follow one among 23 bijective transformation rules, nine symmetric exchanges (X ↔ Y, e.g. A ↔ C) and fourteen asymmetric exchanges (X → Y → Z → X, e.g. A → C → G → A), multiplying by 24 DNA's protein coding potential. Abrupt switches from regular to swinger polymerization produce chimeric RNAs. Here, human mitochondrial proteomic analyses assuming abrupt switches between regular and swinger transcriptions, detect chimeric peptides, encoded by part regular, part swinger RNA. Contiguous regular- and swinger-encoded residues within single peptides are stronger evidence for translation of swinger RNA than previously detected, entirely swinger-encoded peptides: regular parts are positive controls matched with contiguous swinger parts, increasing confidence in results. Chimeric peptides are 200 × rarer than swinger peptides (3/100,000 versus 6/1000). Among 186 peptides with > 8 residues for each regular and swinger parts, regular parts of eleven chimeric peptides correspond to six among the thirteen recognized, mitochondrial protein-coding genes. Chimeric peptides matching partly regular proteins are rarer and less expressed than chimeric peptides matching non-coding sequences, suggesting targeted degradation of misfolded proteins. Present results strengthen hypotheses that the short mitogenome encodes far more proteins than hitherto assumed. Entirely swinger-encoded proteins could exist.

## Highlights

• Chimeric peptides are translated from contiguous regular and swinger RNA
• They are 200x rarer than mitochondrial swinger peptides
• Chimeric peptides integrated in regular mitochondrial proteins are downregulated
• Contiguous regular parts are matched positive controls for swinger parts
• The last point validates results beyond other statistical tests for robustness

## Graphical abstract

**Figure:**
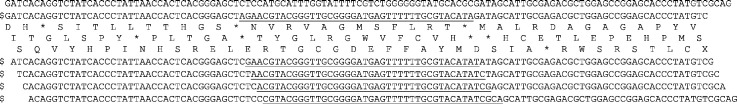

## 1 Introduction

Mitochondrial genomes apparently compensate for their reduced size by cumulating multiple functions for single sequences [12], [14]. For example, tDNA, DNA templating for tRNAs, probably functions also occasionally as light strand replication origin [79], [80], [83], [106], [107], [108], [110]. The complementary strand of tDNA has similar secondary structure formation capacities and might template for additional functional tRNAs with anticodons usually corresponding to the inverse complement of the tRNA's regular anticodon [7], [81], [82], [84]. Mitochondrial tRNA sidearm loops might also function as anticodons, potentially increasing further mitochondrial anticodon repertoires [90], [95]. Translation of stop codons also increases protein coding repertoires [81], reassigning stop codons to amino acids [26], [84], [85], [86], [87], [91], [7], [11], [98].

These various mechanisms expand protein-coding potentials of DNA/RNA sequences. Multifunctional sequences, as suggested for tRNA synthetase genes [53], [69], [70] are presumably relicts of ancient, short protogenomes, plausibly consisting of ancestors of ribosomal RNAs [72], [73], [111], where sequence multifunctionality was probably essential. Presumably, alternative codings are relicts of mechanisms that increase sequence multifunctionality.

### 1.1 Swinger polymerization

A further little known phenomenon increases DNA's protein coding repertoire: nucleotide polymerization that systematically exchanges nucleotides. This alters gene and mRNA coding properties. Assuming this phenomenon enables to detect homology relationships of otherwise ‘orphan’ DNA and RNA sequences. The homology of these orphan sequences had not been determined because these apparently orphan sequences are so much transformed as compared to their ‘parent’ homologue that homology is undetectable without assuming a systematic exchange between nucleotides, but becomes obvious after taking the systematic exchange(s) into account. These transformations consist of systematic exchanges between nucleotides during DNA or RNA polymerization, producing so-called swinger sequences.

The first described swinger RNAs were from vertebrate mitogenomes, and correspond to a 3′-to -5′ inversion, without complementing, of the homologous, template sequence [88], [92], also called ‘reversing’ transformation [27], [28]. When considering a specific sequence, this transformation follows the swinger rule A ↔ T + C ↔ G (bijective transformation rule π9 according to the annotation system in [58]) of the negative strand of the specific sequence, one among nine systematic symmetric exchanges, of type X ↔ Y, e.g. A ↔ C [93]. Fourteen asymmetric exchanges exist, of type X → Y → Z → X, e.g. A → C → G > A [94]. About hundred mitochondrial transcripts corresponding to one of these 23 swinger types have been detected within the human EST database of GenBank, with about twice as many from the nine symmetric exchanges than from the fourteen asymmetric exchanges.

Swinger RNAs matching eleven exchange types were detected within GenBank's EST database, six symmetric, and four asymmetric transformations. Most of these swinger RNAs (obtained by classical Sanger sequencing) are longer than 100 nucleotides and have > 90% similarity with the mitogenome if the swinger transformation is assumed over their complete length [90], [91]. All 23 swinger types exist in the human mitochondrial transcriptome among short reads produced by RNA seq (Illumina) ([103], data from [30]). Abundances of different swinger types as estimated from GenBank's ESTs (sequenced by classical methods) and next generation massive sequencing (RNA seq) are overall congruent (i.e. Figure 2 in [103]). This congruence between swinger RNA abundances is remarkable for two reasons: first because comparable results are obtained by two independent methods (Sanger versus next generation sequencing); and second because biological samples differ (not the same cells/mitochondrial lines were analyzed). This suggests that mitochondrial swinger transcription is general to mitochondria, not tissue- or line-specific.

Hence sequences potentially template for 23 swinger transformed versions, increasing considerably the potential coding density of any sequence. Swinger DNA was also detected for nuclear and mitochondrial genes [96], [97], especially ribosomal RNAs [99], but for now only according to swinger rule A ↔ T + C ↔ G. Swinger sequences detected in Genbank originate from numerous independent research projects and laboratories, only this author describes them as swinger-transformed.

### 1.2 Swinger versus chimeric RNAs and peptides

Some detected sequences are not entirely swinger-transformed, sequences contiguous to the swinger sequence match the untransformed, contiguous DNA template, and hence are regular RNA [100]. These RNAs transcribed partly by regular, and partly by swinger transcriptions, are termed chimeric RNAs [100]. The transition from one to the other part is frequently abrupt, suggesting sudden switches in the polymerization mode of the same polymerase.

Analyzes here search for peptides matching translation of such chimeric RNAs, where contiguous parts of the peptide are translated from regular and swinger parts of the sequence. These peptides are also considered chimeric, and differ from previously described swinger peptides [103] because the latter are only translated from swinger-transformed RNA, while chimeric peptides would be transcribed from RNA that is in part regular, and in part swinger-transformed.

**Fig. 1:**
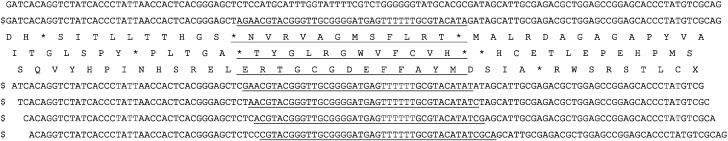
Example of running windows reduced to 120 nucleotides for illustration purposes, for the human mitochondrial genome, and swinger transformation of the mid-third 40 nucleotides. First row: regular genomic sequence; rows starting by $ are the first 5 running windows, with the mid third swinger transformed according to swinger rule A ↔ C + G ↔ T (as an example). Running windows used for actual analyses are 270 nucleotides long, and transformed according to each of the 23 swinger transformations, along the same principles as shown above for 120 nucleotides. The three peptides translated from the first running window sequence are also indicated, stops codons are translated here as ‘*’. Analyses searching for mass spectrometry data matching these predicted peptides consider the possibility that any amino acid is integrated at stops (19 possibilities, merging leucine and isoleucine, which are undistinguishable by the sequencing technique used here, because their molecular weights are identical). This means that for a single running window sequence after a single swinger transformation, 3 × 19 = 57 hypothetical peptides are considered. This number is doubled to 114 considering the inverse complement sequence, and multiplied by 23 considering all 23 potential swinger transformations. Hence 2622 peptides are translated from the 23 swinger transformations of each running window sequence indicated by $. This running window structure enables detection of chimeric peptides where the regular part is translated from the 5′, as well as from the 3′ side of the swinger part.

The principle according to which chimeric sequences are produced is shown for a specific 120 nucleotides long sequence of the human mitogenome (Fig. 1). The mid-forty nucleotides are swinger transformed according to swinger rule A ↔ C + G ↔ T (second sequence in Fig. 1). Swinger RNAs consist solely of swinger-transformed regions such as the underlined transformed regions. Chimeric RNAs have at least one of the contiguous, untransformed (5′ and/or 3′) parts. Swinger peptides are solely translated from swinger-transformed sequences (such as the underlined sequence in Fig. 1). Chimeric peptides are translated from a sequence that stretches over a regular and a swinger-transformed RNA region. Only a minority of detected RNA sequences bearing swinger transformations are chimeric, most follow in their entirety a given nucleotide exchange rule [90], [91], [100]. Detection of chimeric peptides would be evidence independent of previous descriptions of swinger peptides (entirely encoded by swinger-transformed RNA [103]) for translation of swinger-transformed RNA. Much fewer chimeric human mitochondrial RNAs [100] than entirely swinger-transformed human mitochondrial RNAs [90], [91] have been detected. Hence I expect to detect fewer chimeric peptides than for previous analyzes searching for entirely swinger-encoded peptides.

### 1.3 Swinger polymerization by regular polymerases?

Swinger polymerizations could result from unusual polymerization modes by regular polymerases because the principle of swinger polymerization does not differ from that of point nucleotide misinsertions. The difference is in the systematic change in templating rules, from f(A,C,G,T) = (A,C,G,T/U) (regular DNA replication/transcription), to a different rule, e.g. A ↔ C, which can also be annotated as f(A,C,G,T/U) = (C,A,G,T/U), stressing its systematic, rather than punctual nature. It seems plausible that point nucleotide misinsertions are due to switches to unstable, unusual conformations of polymerases, lasting the time of a misinsertion. Hypothetically, these unstable, misinsertion-inducing conformations are occasionally stabilized, so that the nucleotide exchange corresponding to that misinsertion occurs systematically along the sequence stretch polymerized while the polymerase is in that unusual conformation, producing a swinger DNA/RNA. Swinger RNAs are for now the only evidence indicating the existence of such unusual, stabilized polymerase states.

This hypothesis on polymerase conformations yields two testable predictions. The first prediction is that biochemical parameters experimentally estimated for point misinsertions by polymerases predict properties of swinger sequences. In this respect, the affinity (Km) and Vmax of each of the twelve misinsertions, and the four regular insertions, as determined by Lee and Johnson [46]) (therein Table 1), were used to predict abundances of swinger RNAs. Indeed, these experimental kinetic parameters predict several properties of swinger RNAs [58], [93], [94], strengthening the hypothesis that regular polymerases are responsible for swinger polymerizations, by switching to unusual, stabilized ‘swinger’ conformations, similar (or even identical) to conformations causing point misinsertions, but lasting longer.

The second testable prediction of the hypothesis is that the same polymerase produces regular and swinger-transformed sequences. Hence occasionally, polymerases switch in the midst of replication/transcription, so that part of the sequence follows regular templating rules, and the other, contiguous part, is swinger transformed according to one among the 23 swinger rules. The fact that there are far more RNAs that are entirely swinger transformed than chimeric RNAs [100] suggests that such switches during polymerization are rare, and usually occur before or at the onset of polymerization.

The 16S rRNA gene in the complete mitogenome of *Kamimuria wangi* is a swinger transformed A ↔ T + C ↔ G DNA sequence, embedded within an otherwise regular insect mitogenome [99]. The reasons why until now only DNA matching this A ↔ T + C ↔ G swinger transformation has been detected, remain unknown. This A ↔ T + C ↔ G exchange rule is also common among chimeric (part regular, part swinger) RNAs [100].

Peptides encoded by chimeric transcripts are detected for the first time here. An example of three peptides and the corresponding DNA, chimeric RNA is described in Fig. 1. Underlined parts are translated after swinger-transformation of the transcribed RNA sequence. When a detected peptide corresponds only to all or part of the underlined amino acid sequence, this peptide is considered a swinger peptide, as the peptides described in an earlier publication [103]. When the detected peptide encompasses part of the underlined, and part of the contiguous amino acid sequence(s) that are not underlined in Fig. 1, the peptide is considered chimeric, because translated from untransformed RNA (the part that is not underlined in Fig. 1), and from RNA that is swinger translated (underlined part in Fig. 1).

### 1.4 Previously detected swinger RNA

An anonymous reviewer suggested to add, for reader convenience, explanations on how swinger sequences described in previous publications had been detected in GenBank. These methods are not described in the Materials and methods section, as this would be inadequate and confusing: neither results nor analyses beyond those described in earlier publications on RNA were done in the context of the presently described proteomic analyses. The aim here is (chimeric) peptide detection. Following descriptions are only for the convenience of potential readers.

In a first step, the 23 swinger versions of the human mitogenome were transformed *in silico*. This means that for swinger transformation A ↔ C (as an example), all As in the human mitogenome are replaced by the ‘replace’ function in the software Word by ‘X’. Then all Cs are replaced by A. The last step is to replace all Xs by C, producing a hypothetical, A ↔ C swinger transformed human mitogenome. Similar procedures produce all 23 possible swinger versions of the mitogenome. Each of these is then analyzed by BLASTn [2]. Two types of analyses have been done. The first analyzes (publications by Seligmann on swinger sequences prior to 2016, from 2012 on) search for alignments between the swinger transformed mitogenome and various sequence databases in GenBank, using standard default megablast parameters. This resulted in detecting long, highly similar sequences, as described in [88], [92], [93], [94], for example. Such searches do not yield alignments with nuclear chromosome sequences, but detect about 100 ESTs (expressed sequence tags). The length of the alignments (> 100 nucleotides) and the similarity with the hypothetical swinger-transformed mitogenome versions (> 90%), as these were previously presented (Table 1 in [88], [92], and Table 2 in [93], [94]), are not compatible with randomly obtained results (as tested by simulations based on randomly shuffled swinger mitogenomes in [96] (therein Section 2.2.3.)).

**Fig. 2:**
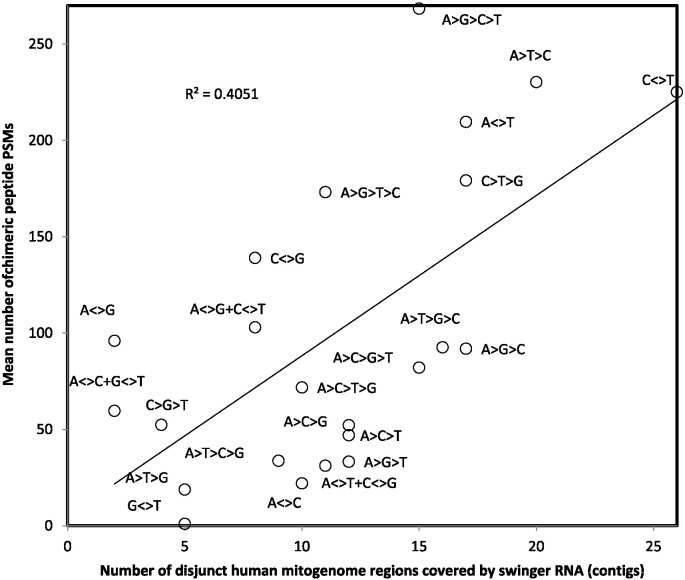
Mean number of PSMs detected for chimeric peptides, as a function of the number of disjunct human mitogenome regions covered by swinger RNA (RNA data from [102]). The positive association indicates the expected causal link between swinger RNA and chimeric peptides.

These long EST swinger sequences were then confirmed by sequences detected within sequence read archives (SRA) of the human transcriptome published by Garzon et al. [30]), GenBank SRA entries SRX768406–SRX768440. For these analyses pertaining to short RNA reads (50 nucleotides, RNA seq, Illumina), BLASTn searched for ‘somewhat similar sequences’, using default search parameters, and detected swinger reads as these are described in [103] (therein supplementary data). These results on short swinger reads converge with those obtained by the first, EST-focused search ([103], therein Figure 2). Using the same search tool and criteria as used for the RNA seq reads, the 23 swinger-transformed mitogenome versions also align with nuclear chromosome sequences (they did not align with human nuclear chromosomes when using megablast as for the EST search).

### 1.5 Nuclear origins of swinger sequences (numts)

Alignments detected with relaxed criteria (BLASTn, see previous section) between swinger mitogenome sequences and each short reads and nuclear chromosome sequences suggest the possibility that swinger reads (but not swinger ESTs) could originate from the cytosol. To some extent, this is not relevant to the main issue at stake, the very existence of swinger polymerizations, but could be relevant because the very large human nuclear genome could by chance be the origin of these alignments, due to its size.

In addition, mitogenome copies (called numts, [51]) are inserted within nuclear chromosomes [36]. Because nuclear copies of regular mitogenomes exist, their occurrence for swinger-transformed mitogenomes is plausible, and would consist in itself a possible independent confirmation of the existence of swinger sequences, as previously discussed [103]. In addition, the possibility of swinger transcription of regular numts in the nucleus can't be ruled out.

Previous analyses [103] showed that the majority of detected swinger reads have mitochondrial origins. On average, alignments between swinger-transformed mitogenomes and RNA seq reads have higher identity percentages than between the same swinger mitogenome sequences and nuclear chromosome sequences. This is the case for a statistically significant majority of comparisons of identity percentages obtained between RNA reads and the swinger-transformed mitogenome, versus that between the same swinger-transformed mitogenome region and nuclear chromosome sequences. This suggests that most potential swinger numts diverge from their ancestral mitogenomic sequence, and that most RNA reads aligning with swinger-transformed mitogenomes have mitochondrial origins, because the swinger-transformed mitogenomes resemble on average more RNA reads than putative swinger numt(s) [103]. The ‘Discussion’ below develops these points in relation to potential nuclear origins of chimeric peptides.

### 1.6 Chimeric RNAs due to fusion between different RNAs

The term ‘chimeric’ transcripts has been used in the literature for a different type of RNA than the contiguous regular- and swinger-transcribed RNAs [101]. These other types of chimeric RNAs refer to two or more different transcripts produced each by regular polymerization, on the template of disjunct DNA regions. These RNAs are then fused by natural [60], [132] or artificial reverse-transcription-associated phenomena [131]. These chimeric RNAs differ from the regular-swinger RNAs in the sense that for the latter the transcription process is chimeric (part regular-, part swinger transcriptions), but not in terms of their templating DNA regions, which are contiguous, not disjunct. It is possible that some unknown sequencing artifacts produce some of the detected swinger reads, but the non-random mapping of detected swinger peptides on detected swinger RNA reads, as previously described [103] shows that most swinger reads exist while translation occurs in the cell, and hence are not artifacts.

### 1.7 Swinger polymerization creates new genomic sequences

Another type of analyses detected swinger repeats within the regular mitogenome. Swinger repeats are usually short repeats that can only be detected when taking into account swinger transformations. These short sequences are inserted within the regular mitogenome, suggesting that natural retrotransposition of swinger RNAs produces novel DNA sequences [101]. They are more frequent and longer than expected by chance, and their length is proportional to the probability that the specific swinger transformation conserves circular code signals that presumably maintain the ribosomal translation frame in the gene. The natural circular code is a punctuation code within the genetic code consisting of 20 codons that as a group, have properties that enable protein coding frame retrieval [4], [22], [55], [56], [57]. This indicates that insertion of swinger sequences in the human mitogenome depends on their capacity to integrate protein coding genes without disrupting punctuation that presumably enables ribosomal detection of the coding frame.

### 1.8 Chimeric peptides

Recent analyses show convergent frequencies between swinger RNAs sequenced by classical and next generation (RNAseq) sequencing methods [103]. Hence swinger RNA occurrence is relatively well confirmed by data from independent methods and research teams. Here analyses complement at peptide levels results on chimeric transcripts. The existence of transcripts that are part regular (untransformed), part swinger RNAs, with an abrupt switch between these parts, predicts the existence of ‘chimeric’ peptides matching translation of such chimeric transcripts. Hence MS/MS mass spectra of peptide data (from [33]) previously used to detect swinger peptides [103] are reanalyzed here, using the same methods as by Seligmann [103]), searching for peptides matching in part the translation of the untransformed human mitogenome, and in part the translation of the swinger-transformed, contiguous mitogenome sequence. Chimeric peptides are peptides where swinger-encoded parts of a peptide are contiguous with parts translated from regular RNA. These would be stronger evidence for translation of swinger RNA than previous detections of entirely swinger-encoded peptides because the regular encoded parts function as matched positive controls, directly associated with swinger-encoded parts. In addition, chimeric peptides could suggest that swinger peptides are integrated within otherwise regular proteins, a further small step to understand functions associated with swinger phenomena.

## 2 Materials and methods

The revised Cambridge reference sequence for the human mitochondrial genome (NC_012920, [3]) was cut according to a running window of 270 nucleotides. Analyzes do not account for known mitochondrial polymorphisms, as this would expand analyzes beyond computing powers. The six frames of each of these nucleotide sequences of 270 bases were translated into the corresponding hypothetical peptides according to the vertebrate mitochondrial genetic code, after the 90 nucleotide-long mid-third of that sequence was swinger transformed according to each of the 23 swinger transformations. Hence each running window (around 16,300 in total) is represented by the six peptides translated from each of its 23 partly swinger-transformed versions (6 frames × 23 swinger versions = 138 hypothetical chimeric peptides for each of the 16,300 running windows). The window length of 90 codons/amino acids is designed to match the length of the longest (non-chimeric) peptides (up to 40 amino acids, [103]) previously detected in this dataset [33]. All translated hypothetical peptides are used by Thermo Protein Discoverer to predict a theoretical mass spectrometry distribution, which is matched with observed MS/MS mass spectrometry data from Gueugneau et al. [33]).

Stops are translated as ‘X’, which Thermo Proteome Discoverer considers by default as leucine/isoleucine (these have equal masses, and are indistinguishable by mass spectrometry). Peptides including stops are duplicated 18 times, replacing ‘X’ by one of the 18 remaining amino acid species, excluding leucine and isoleucine. Hence predicted peptides include the possibility that any amino acid could be inserted at stops. Analyses assume that all stops in a single predicted peptide are translated by the same amino acid. Hence the 138 peptides for a single window of 270 nucleotides, if it includes at least one stop (the majority of cases), are represented 19 times, inserting X and each of the remaining amino acids at stops (19 x 138 = 2622 chimeric peptides). In total, approximately 42.7 million hypothetical chimeric peptides were tested.

Consensus searches were handled with the Sequest (Thermo Fisher Scientific, Illkirch) algorithm with the following mass tolerances: Parent = 1 Da and Fragment = 0.5 Da (monoisotopic masses). Fixed carbamidomethyl (C) and variable Oxydation (M) modifications were activated, as well as the lysine → pyrrolysine modification, and only one missed trypsin cleavage was allowed. False discovery rate was estimated against a reverse decoy database using the Percolator algorithm. No protein grouping was allowed since the database only contained non redundant entries. Peptides with false discovery rate q < 0.05 and score Xcorr > 1.99 were considered identified. The score Xcorr is a likelihood of match between expected and observed MS/MS data that is unaffected by peptide length. Further explanations on peptide detection and characterization by the software are given in the Discussion. Observed mass spectra were compared separately to predicted peptides 19 times, each time inserting a different amino acid at stops. Here analyzes test the existence of a specific group of peptides, namely chimeric peptides. The false discovery rate q is adapted to such populations of detected items [41]. Results also indicate the posterior error probability PEP, an estimate of detection error specific to each individual peptide, which might be useful in the future, when analyzes focus on specific peptides, rather than on a population of peptides. Results are not analyzed according to this criterion more adapted to studies focusing on specific individual peptides.

## 3 Results

### 3.1 Chimeric peptides

Analyses detect according to the filtering criteria 1301 chimeric peptides, among approximately 42.7 million chimeric peptides produced by combinations of stop codon-amino acid insertions, swinger transformations and frames for the running window of 270 nucleotides (illustration in Fig. 1). Hence chimeric peptides are detected for approximately 3 among 100,000 hypothetical chimeric peptides. This is 200 times less than the rate of detection for ‘regular’ non-chimeric swinger peptides, using the same criteria and the same data, approximately 6 per 1000 predicted swinger peptides [103].

Previously detected chimeric human mitochondrial RNAs are about 3% of all RNAs detected with at least some swinger part. Part of the discrepancy between chimeric RNA versus peptide detections probably results from the fact that proteomic analyses only considered abrupt switches between regular and swinger parts of peptides. Blast analyses detecting RNAs are not limited by this consideration, and can detect RNAs where the switch is not abrupt: in a transition sequence between regular and swinger transformed sequences, nucleotides seem random. Hence for practical reasons, detection of chimeric RNAs encompasses more possibilities than chimeric peptide detection, explaining lower rates of chimeric peptide detection (relative to rates of swinger peptide detection); these are lower than rates of chimeric RNA detection (relative to swinger RNA detection rates).

Here we focus specifically on chimeric peptides for which each regular and swinger parts have more than 8 amino acids. This is because considering 19 different amino acid species (merging leucine and isoleucine), the e value for 42.7 million potential chimeric peptides is about 0.0001 for amino acid sequences of 9 residues (42,000,000 × 1/19^− 9^). Hence the match of each regular and swinger part of the detected peptide with the predicted chimeric peptide is unlikely to be due to chance, as estimated by this approximate e value.

This restricts the sample of 1301 detected chimeric peptides to 186 chimeric peptides of at least 18 residues, from various swinger transformations and stop-amino acid insertions (Table 1). Among these 186 chimeric peptides, the regular-encoded part of the peptide corresponds to the 5′ part of the peptide for 41% of the 186 chimeric peptides. This means that a statistically significant majority of chimeric peptides (two tailed sign test, P = 0.0061) correspond to the 5′ translation of swinger RNA and 3′ translation of regular RNA. Note that this statistically significant bias could not occur if detected chimeric peptides were due to random detection artifacts, strengthening the suspicion that results reflect a biological reality. Hence 41% of chimeric peptides reflect translation of regular transcripts that switch at a given point to swinger transcription. Frequencies and mean lengths of chimeric peptides, for each swinger type (Table 2) show that the regular (non-swinger) part of the chimeric peptides is on average slightly longer than the swinger part, though this difference is not statistically significant.

### 3.2 Swinger peptides and chimeric peptides

Note that chimeric peptides, due to their part that matches translation of regular transcripts, differ in mass spectrometry properties from peptides entirely translated from swinger RNA, even if these have the exact same swinger sequence. Hence detection of chimeric peptides with swinger parts overlapping previously detected ‘regular’ swinger peptides (as the swinger peptides described by [103]) would be strong, independent methodological confirmation that positive results are not artifacts. Indeed, the swinger parts of eight chimeric peptides in Table 1 overlap with one of the 263 previously described swinger peptides [103]. These previously described swinger peptides cover on average 1.1% of the swinger-transformed mitogenome, expecting approximately 2 overlaps with chimeric peptides in Table 1 if no association exists between the two independent analyses. This means that chimeric peptides map on previously described swinger peptides 4 times more frequently than expected. This association between two independent searches confirms that results are not false positive matches between the mass spectrometry data and some predicted hypothetical chimeric peptides among a very large number of predicted hypothetical chimeric peptides. In addition, note that even if detected swinger and chimeric peptides correspond to the same swinger region, the corresponding MS/MS mass spectra differ, because for chimeric peptides mass spectra include also the adjacent residues translated from regular, untransformed RNA, while for swinger peptides, mass spectra do not include the latter residues. This non-random correspondence between swinger peptides and swinger parts of chimeric peptides suggests that translation of swinger RNAs is not random, and probably specific to some mitogenome regions.

### 3.3 Swinger RNA and chimeric peptides

Previously detected swinger peptides preferentially map on human mitogenome regions covered by independently detected swinger RNAs [103]. Their numbers increase with numbers of detected swinger transcripts. These positive associations between swinger RNA and swinger peptides can also be expected for chimeric peptides described in Table 1, Table 2. Such associations would confirm that the detected chimeric peptides actually exist, because they would match two independent material evidences, peptides, and RNA fragments.

The mean number of PSMs (peptide spectrum matches) for chimeric peptides increases as a function of the number of human mitogenome regions covered by swinger RNA (also called contigs), for the swinger type corresponding to the swinger part of the chimeric peptides (Fig. 2). Swinger transcriptomic data are from Seligmann [103]). Chimeric peptides presumably reflect translation of chimeric RNAs, along part regular, and part swinger transcription rules. Hence amounts of chimeric peptides should reflect numbers of possible transitions between regular and swinger RNAs, estimated by the number of swinger contigs previously described by Seligmann [103]). Indeed, a positive association between PSMs of chimeric peptides and swinger RNA contigs exists (r = 0.64, one tailed P = 0.0006), strengthening confidence in the validity of results, and corresponding with previous results for swinger peptides [103]. Note that similar correlation analyses for numbers (not PSMs) of detected chimeric peptides do not yield statistically significant associations with contig numbers.

The swinger part of 8 chimeric peptides (marked by * in Table 1) maps on human mitogenome regions also covered by the adequate type of swinger RNA (six swinger types, two matches for A → G → T → A and A → C → G → T → A, and one match for each A → G → C → T → A, A → G → T → C → A, A → T → C → G → A, and A → T → G → C → A swinger transformations). Considering the overall mitogenome coverage by swinger RNAs (on average 2.6% of the genome), lack of association between swinger RNAs and the swinger part of chimeric peptides would expect 4.76 matches across all 23 swinger transformations, with 0.21 peptides for the average swinger transformation. This predicted number for specific swinger transformations was always < 0.5 peptides. Detecting at least one match for six among 23 swinger types, when less than 0.5 are expected for all 23 swinger transformations has P = 0.022 according to a two-tailed Fisher exact test. This indicates that chimeric peptides associate with detected swinger RNA, though this association is weaker than the previously described association between swinger RNA and swinger peptides [103].

### 3.4 Chimeric peptides: strong validation of swinger sequences

Chimeric peptides are in terms of confirmation of swinger polymerization only secondary evidence, because peptides are translated from RNA, as compared to previous descriptions of swinger RNAs and chimeric swinger RNAs [100], which directly result from swinger polymerization. This point is also valid for swinger peptides. However, detection of (numerous) peptides matching translation of contiguous parts of the mitogenome, where one part reflects regular transcription, and the other swinger transcription, is a strong methodological confirmation for swinger phenomena and associated translation into peptides, which is not implied by the detection of ‘pure’ swinger peptides. This is because the non-swinger part of the peptide is a positive control paired to its contiguous swinger part. Hence in addition of describing a further aspect of the biological phenomenon of swinger polymerizations, chimeric peptides are also a further validation of the phenomenon's existence.

### 3.5 Chimeric peptides integrated in regular proteins?

An important question associated to swinger sequences is their function: among others, do they code for functional proteins, and are swinger peptides integrated into regular, perhaps functional proteins? A reanalysis of Table 1 yields a first insight into these important questions. The regular (non-swinger) part of eleven peptides matches the sequence of six among thirteen known, regular, mitogenome-encoded proteins. Their swinger parts correspond to the translation of the contiguous swinger transformation of these genes, along nine (four symmetric, and five asymmetric) systematic nucleotide exchange rules (Table 3). Note that up to three chimeric peptides are detected for two large mitochondrial proteins (cytochrome c oxidase I and NADH:ubiquinone oxidoreductase subunit 5). It is plausible that such peptides are integrated within complete proteins. These sequence alterations could modulate (or not) the regular function of the protein, and not necessarily impair function.

These 11 chimeric peptides integrated in regular proteins represent 5.9% of all 186 detected chimeric peptides. Considering that regular mitochondrion-encoded proteins have a total length of 3789 amino acids, the regular proteins represent 11.43% of the total number of amino acids that could be translated from the positive and negative strands of the human mitogenome. This means that chimeric peptides embedded within regular coding sequences are half as frequent as expected (5.9 versus 11.43%). This principle is further strengthened when examining the number of PSMs (number of identified peptide spectra matching a hypothetical peptide) for these 11 regular-protein-integrated chimeric peptides, as compared to the mean number of PSMs for all chimeric peptides detected for that swinger transformation: their PSMs is in all but one case (peptide 80 in Table 1) lower than the mean PSMs of other chimeric peptides for that swinger transformation. Hence chimeric peptides within regular proteins are rarer, and less expressed (as far as PSMs numbers can be trusted to reflect peptide abundances), than chimeric peptides translated from non-coding sequences, and non-coding frames of regular protein coding genes.

### 3.6 The natural circular code and swinger RNA, peptides and chimeric peptides

An anonymous reviewer suggested examining whether properties of chimeric peptides can be predicted from frameshift error-correcting properties of the natural circular code. Indeed, abundances of detected swinger RNAs in GenBank's EST database are proportional to reading frame retrieval (RFR) after swinger transformation of the natural circular code [58]. In this context, RFR, which estimates the capacity of the natural circular code to retrieve the protein coding frame, is calculated for the 20 codons that form the natural circular code, after each of the 23 swinger transformations: some codons belonging to the natural circular code are transformed into another codon included in the natural circular code, meaning that this property is invariant in relation to that codon and swinger transformation. RFR estimates this across all 20 codons of the natural circular code, for each swinger transformation. The length of swinger repeats in the human mitogenome is proportional to the RFR of the swinger transformation [101], which suggests that RFR affects insertion rates of swinger repeats in protein coding regions, and hence could also affect chimeric peptide production.

The association between RFR and swinger RNA abundances for EST sequences occurs also for mitogenome coverage by swinger RNA reads sequenced by RNAseq in the transcriptome by Garzon et al. [30]) (Pearson correlation coefficient r = 0.528, one tailed P = 0.005). For swinger peptides as described by Seligmann [103]), the mean number of PSMs also increases with RFR (r = 0.364, one tailed P = 0.044). This positive association between mean PSMs numbers and RFR is also detected for chimeric peptides from Table 1 (r = 0.367, one tailed P = 0.043). These two results are independent, also because mean PSMs of swinger and chimeric peptides are only weakly correlated (r = 0.24, P > 0.05). Hence detections of chimeric and swinger peptides are proportional to extents by which swinger transformations conserve natural circular code ‘frame’ punctuations. Note that RFR, as mitogenome contig numbers in a previous section, associate with mean PSMs, rather than numbers of detected peptides, suggesting that in the context of these specific data, PSMs are better quantitative estimates than other variables.

## 4 Discussion

### 4.1 Statistical validity of peptide detections by mass spectrometry

An anonymous reviewer of a previous version indicated that detection of peptides with masses approximately matching the numerous possibilities produced by translation of all potential chimeric RNAs could be due to chance, due mainly to the large number of hypothetical chimeric peptides. Indeed, considering all 19 possible amino acids inserted at stops introduces a ‘fudge’ factor that enables adapting many hypothetical peptides to an actual fragment with a similar mass. Note that 28 among 186 (15%) detected peptides lack stops, invalidating this argument for several detected chimeric peptides. Independently of this, there are three reasons why this important point does not invalid the remaining results on chimeric peptides presented here. This is first because mean chimeric peptide PSMs converge with corresponding swinger RNA contig numbers, an independent type of data unrelated to the problems of proteomic analyses, already discussed above.

The other two points relate to the nature of the MS/MS mass spectrometry analyses themselves. The factor ‘detection by chance’ is integrated into the detection software used by Thermo Proteome Discoverer. The software compares the match between the mass spectrum of the actual fragment and the predicted mass spectrum of the hypothetical peptide, and its match with a dataset of decoy (false, negative controls) predicted peptides. The q value estimates the false detection rate (FDR, see explanations by [41]) of a peptide based on comparing matches by the actual predicted peptides and the decoy peptide database. This q is a probability of detection corrected for the false detection rates within the population of positive results (classical P values consider the whole population of statistical decisions, not only the subpopulation of positives). Hence the reported detections account for matches due to chance, considering the various parameters of the samples analyzed/compared, among them in particular sample sizes.

The third point relates to the nature of the statistic whose distribution is used to evaluate the above mentioned q (FDR). It is Xcorr, the cross correlation of the goodness of fit between the experimental peptide fragments and theoretical mass spectra. This integrates fits with each b and y ions, which correspond to asymmetry in the physical fragmentation of peptide bonds within the detected peptide, resulting into shorter peptide subparts: b ions occur when the residue's N-terminal is charged, y ions when the C-terminal is charged. Hence the match between the observed and the predicted peptide is not based solely on the similarity between their total masses, but also on fit between distributions of masses of sub-fragments of the (expected and observed) peptides, and this separately for b and y ions. The Xcorr statistic accounts, in addition to peptide size, for the number of matching masses of such sub-fragments. This allows inferring more precisely the residue sequences in the peptide, and means that peptide detection is not based only on a single measure, its total mass, but also on the mass of several subfragments.

In this context, the peptide ACD can function as a simplified example. Its mass corresponds to six possible peptides, ACD, ADC, CAD, CDA, DAC and DCA. Hence if ACD results from translation of swinger RNA, one can't assert that the observed mass is due to this peptide rather than any of the other five possibilities. However, Xcorr also considers the masses of subfragments of this peptide. Detection of a subfragment matching the mass of AC excludes four among the six possible peptides. A fragment matching the mass of CD matches only two peptides. If both subfragments AC and CD are detected, the characterization of the peptide ACD can be considered as assessed.

In addition, this process is done separately for b and y ions, because mass spectrometry analyses are in principle sufficiently precise to distinguish between these ions (remember that the precision of 0.5 Da of the analyzed data means a precision of half the mass of a hydrogen atom, which is also far less than the difference between amino acids with similar molecular masses). Hence Xcorr integrates information from both b and y ions, evaluating whether that information is congruent with the observed data. This procedure, coupled to q values based on comparisons of the Xcorr distribution obtained for negative controls (decoy peptides), renders detections relatively robust, despite fuzzy factors. In fact, large numbers of predicted peptides are necessary to estimate properly the distribution of random Xcorrs. The last point stresses that q (as P) values account for numbers of predicted peptides.

### 4.2 Confirmation of chimeric peptides by Waters technology

An anonymous reviewer suggested to confirm the existence of chimeric peptides by additional, independent mitochondrial proteomic data. In this context, I focused on another analysis of trypsinized human mitochondrial peptides [1], extracted by a more up to date MS/MS technology (Waters, Milford, MA, http://www.waters.com). This technique yields more accurate mass estimates than the method used by [33] (0.5 Da for the latter versus 5 ppm for the Waters method, hence about 10 × more accurate estimates).

Analyses of the twelve samples from Alberio et al. [1]) by the software PLGS yield relatively few hits matching chimeric peptides considering only peptides where each regular and swinger-encoded parts are each at least nine amino acids long. One peptide matches significantly according to PLGS a chimeric peptide whose swinger part (underlined) matches swinger transformation A → T → G → A, LVSASVEMNQQQVPGSAGR (the regular part are residues 4228–4237 translated from the third frame of the negative strand of the human mitogenome). The other peptide detected in these data has a swinger part that matches transformation C → G → T → C, SAAAARAGSACCLTSTAVTDRNLNTTF, the regular-encoded part corresponds to COX1, residues 211–219 in that regular mitochondrion-encoded protein.

Hence a different technology detects within independent mitoproteomic data peptides matching translations of chimeric RNAs, with one part regular, the other swinger transformed RNA. Hence, at least qualitatively, these independent data and technology confirm the existence of chimeric peptides and their integration in regular mitochondrial proteins. A more detailed description of ‘regular’ swinger peptides (meaning peptides entirely coded by swinger transformations of the mitogenome (unlike chimeric peptides that are in part regular-, in part swinger-encoded)) detected in the data from Alberio et al. [1]) will be presented elsewhere.

These results from data by Alberio et al. [1]) are too scarce to indicate whether chimeric peptides are produced according to a non-random profile. However, the non-random convergence between chimeric and entirely swinger peptides (detected in the same dataset from [33]) noted in a previous section in Results is in itself an indication that swinger-encoded peptides or parts of peptides are non-randomly produced.

### 4.3 Nuclear mitogenome copies

Previous transcriptomic analyses that detected non-canonical RNAs transformed according to systematic rules, such as deletions of mono- and dinucleotides after each transcribed trinucleotide (producing delRNAs, [102]), and swinger transformations [103], included controls that account whether the transformed mitogenome versions match nuclear chromosome sequences: mitogenome analyses are frequently contaminated by such chromosomic pseudogenes [9], [10], [48], [49], [50], [51], [62], [66], [67], [123], [124], [125], [133], [134].

These previous analyses blasted the swinger-transformed mitogenome versions versus the (regular) human nuclear chromosomes. For transformed mitogenome regions aligning with both transcriptomic reads and chromosomes, similarities between the transformed mitogenome and the RNA contigs were compared with the corresponding similarities between the same transformed mitogenome region and the chromosomes. For each del- and swinger RNAs (non-canonical RNAs), similarities with RNA contigs were greater than those with chromosome sequences in significant majorities of cases [102], [103], as already discussed above for swinger RNA reads.

These results indicate two major issues. First, overall, RNA contigs result from non-canonical transcriptions of the mitogenome, the point that was being tested. Second, the observation that chromosome sequences match transformed versions of the mitogenome suggests that chromosomes include inserts of mitogenomic origins that were transformed according to systematic rules. The observation that these are on average less similar to the transformed mitogenome than RNA contigs suggests that these transformed mitochondrial sequences inserted in nuclear chromosomes mutated apart from the original sequence, as expected for inserts lacking function in the cell's nucleus [16], [24], [29], [34], [35], [36], [38], [40], [52], [54], [63], [65], [71], [77], [113], [116], [117], [118], [119], [126], [128], [129].

### 4.4 Peptides translated according to nuclear or vertebrate mitochondrial genetic codes

Similar-minded analyses at the peptide level can test whether chimeric peptides in Table 1 were translated according to the human mitochondrial or the nuclear genetic codes. For that purpose, the regular and swinger transformed versions of the human mitogenome were translated according to the standard genetic code, which differs from the vertebrate mitochondrial genetic code by the reassignment of codon ATA from Met to Ile, of TGA from Trp to stop, and AGR from stop to Arg [23]. These four codons are 6.25% of all 64 codons.

Each swinger- and regular-encoded part of detected chimeric peptides has at least 9 amino acids. Hence the probability of detecting chimeric peptides that would have identical sequence according to both genetic codes is (1 − 0.0625)^− k^, where k is the total length of the peptide. This principle is applied to the chimeric peptides in Table 1 so as to calculate the predicted number of peptides, for each size category, that is expected to match translation according to both nuclear and mitochondrial genetic codes. Lengths of chimeric peptides in Table 1 range from 18 to 42 residues, a total of 24 length categories. The observed number of chimeric peptides compatible with translation according to both genetic codes (in total 30 among the 186 chimeric peptides) is lower than expected in 16 among 24 size categories. Obtaining this result has P = 0.038 according to a one-tailed sign test. This means that, considering the length of chimeric peptides, there are statistically significantly fewer than expected peptides with sequences compatible with translation according to the nuclear genetic code.

The same principle can be applied to chimeric peptides in Table 1 whose sequences are only compatible with translation according to the mitochondrial genetic code, separately for each the regular- and the swinger-encoded parts. Here, the observed number (54) should be larger than the predicted number, if the sample is biased towards mitochondrion-encoded/translated peptides. Considering that 6.25% of codons differ in codon-amino acid assignments between the two genetic codes, the total expected number of chimeric peptides, considering their size, containing at least one of the 4 codons with coding assignment differing between nuclear and mitochondrial genetic codes is 35.97. This number is far lower than the observed 54 according to a chi-square test (P = 0.0027). Hence chimeric peptides with sequences compatible only with translation according to the mitochondrial genetic code are significantly more frequent than expected. This bias confirms the mitochondrial origin of chimeric peptides in Table 1. The number of peptide length categories where more observed peptides than expected are only compatible with mitochondrial translation is again 16 among 24 length categories, which has P = 0.038 according to a one tailed sign test.

These analyses show that detected peptides are more likely translated according to the mitochondrial genetic code than according to the nuclear genetic code. Note that translation, within the mitochondrion, according to the nuclear code is possible: it potentially depends for some codons upon the presence of cytosolic tRNAs, which could be occasionally imported in mitochondria [21], [32], [39], [44], [75], [76], [78], [112], [114]. However, this rationale is not symmetric: cytosolic translation according to the mitochondrial genetic code is much less probable than the opposite, so that nuclear origins are not compatible with the results obtained.

In fact, whether peptides have cytosolic or mitochondrial origins does not actually affect the main point that is addressed here, which is that these peptides were translated in part from swinger RNA. The same point applies to the potential nuclear (numt) origin of swinger-transformed mitochondrial DNA: independently of the location of the process, detection of chimeric peptides implies that swinger transformations occurred, whether during transcription of the regular mitogenome or nuclear inserts, or during numt insertion, possibly by natural swinger retrotranscription. This does not exclude the possibility that some detected chimeric peptides originate from the cytosol, but stresses the fact that most are mitochondrial, and that this issue is not directly relevant to the fact that swinger RNAs, chimeric RNAs, and corresponding peptides, exist, independently of the question of which cellular compartments produce them.

### 4.5 Few chimeric peptides in regular proteins translated from mitochondrion-encoded genes

Chimeric peptides in Table 3 have regular parts that match sequences of regular mitochondrial proteins encoded by mitochondrial genes. These are about 5% of all 186 detected chimeric peptides. Peptides translated from regular mitochondrial genes represent about 11% of the total length potentially translated from the complete mitogenome, considering all six frames. Hence these 11 chimeric peptides potentially integrated in regular mitochondrial proteins are half as frequent as one could expect. Their PSMs is lower than for other chimeric peptides. These are hence rarer and less expressed than one could expect. Possibly, chimeric peptides integrated in regular proteins perturb proper protein folding. Incorrect folding induces various degradation mechanisms associated with mitophagy [5], [42], [122], which could explain that only few chimeric peptides are detected within regular proteins. These findings are not incompatible with the possibility that at least some swinger transcripts and peptides are functional.

### 4.6 Secondary structure formation by swinger transformed RNA and swinger RNA detection

Secondary structure formation by self-hybridization of DNA/RNA groups bijective transformations into three classes of each eight transformations. These share self-hybridization properties within each class [27], [28]. This means that seven bijective transformations (including A ↔ T + C ↔ G) conserve self-hybridization properties of the original, untransformed sequence. Secondary structure formation by swinger RNA associates with swinger RNA detection [104], but these groupings/properties do not correlate with differences in chimeric or swinger peptide abundances/PSMs (not shown). The issue of regulation of alternative mitochondrial transcriptions, respectively post-transcriptional splicing, in relation to secondary structure formation by transformed RNA [61] remains unclear: a positive association exists between RNA occurrence and secondary structure formation for regular and swinger RNAs, but for transcripts resulting from systematic deletions (delRNAs), a negative association exists between secondary structure formation after deletions and delRNAs [105].

### 4.7 Swinger transformations, RNA–DNA differences (RDDs) and heteroplasmy

Specific non-random point differences occur between DNA and RNA sequences, either due to nucleotide substitutions [47] or inserts/deletions [15], including for human mitochondrial transcripts [8], [37], [59]. These RDDs appear shortly after transcripts exit polymerases [130], suggesting RDDs are due to post-transcriptional edition. The systematic repetition of transformations over long sequence stretches that characterize swinger RNA seem less likely produced by post-transcriptional edition than some unusual stabilized polymerase state, however, at this point, no possibility can be excluded, and potential connections of del- and swinger RNAs with RDDs should be kept in mind.

For the same reason, and by definition, punctual mitochondrial heteroplasmies [45], [74], [115], [121] could not account for swinger parts of chimeric peptides, because these have to be translated from sequences differing from standard mitogenome sequences by far more than punctual nucleotide substitutions. Mitochondrial length heteroplasmies are common (49% of individuals, [64]), and in principle could, by chance correspond to swinger-like inserts in the mitogenome. Considering the seven regions containing length heteroplasmies described by Ramos et al. [64]) (therein table 3), only three among 186 chimeric peptides in Table 1 (peptide numbers 3, 156 and 157) potentially overlap (and this only in part) with these length heteroplasmies. Hence length heteroplasmies map non-randomly on chimeric peptides (3 among seven). Hence some presumed chimeric peptides might be translated from regions presenting length heteroplasmies, but this explanation is compatible with, at most, a small minority of chimeric peptides. Hence heteroplasmy could not explain chimeric peptides.

### 4.8 Translation increasing codon size or transcription systematically deleting nucleotides

It is important to note in the broader context of the discussion of results that further little known mechanisms increase the coding potential of sequences. A different, sometimes tRNA-based mechanism produces an alternative decoding of sequences, that of systematic frameshifting, which expands the codon from three to four (or five) nucleotides [6], called here tetracodons or pentacodons. This could result from systematic ribosomal slippages, a phenomenon that would correspond to programmed frameshifts (e.g. [25], [43]), but occurring systematically, and serially; and/or from translational activities of tRNAs with expanded anticodons [68], [120], [127]. These cases relate to previously described isolated frameshift mutations, interpreted as isolated tetra-, pentacodons.

The hypothesis of an early genetic code based on quadruplets was suggested by Baranov et al. [6]) to solve the problem that the weak triplet codon-anticodon interactions could not occur from a thermodynamic point of view in the absence of ribosomes, especially if these occurred at high temperatures [17], [18], [19], [20]. Molecules as complex as ribosomes probably were absent at proto-life stages. Codon-anticodon interactions between four (or more) base pairs are more stable than those between three base pairs. Symmetry considerations also enable the deduction that the primeval genetic code was based on a subset of 64 quadruplets, called the tesserae, specifically for the vertebrate mitochondrial genetic code [31].

The expanded codon hypothesis is that modern genes include overlapping coding regions that consist of series of tetra- or pentacodons. This hypothesis is compatible with bioinformatic analyses where all eight frames of mitochondrial genes were translated assuming tetracodons. Blast analyses detected alignments between parts of these hypothetical tetracoded peptides and regular proteins in GenBank. Several other analyses based on codon usages in these tetracoding sequences confirm their special coding status, including higher GC contents than in non-tetracoding neighboring mitochondrial sequences. This corresponds to the prediction that tetracoding is an adaptation to translation at high temperature [89]. This point was further confirmed by a positive correlation between predicted tetracoding in lizard mitogenomes and mean body temperature in these lizard species [109]. Accordingly, overlap coding by tetracodons increases with temperature.

At this point, and besides the proven existence of decoding mechanisms for isolated tetracodons, the strongest further evidence for the existence of protein coding regions based on tetracodons is the coevolution between predicted tetracoding regions and the predicted antisense mitochondrial tRNAs with expanded anticodons, which is observed in mammal and *Drosophila* mitochondria [88], [90], [95]. In addition, mitochondrial peptides matching translation of regular and swinger RNAs according to tetra- and pentacodons have been detected [103], as well as translation of delRNAs (or dRNAs), RNAs transcribed while systematically deleting every fourth, or every fourth and fifth nucleotide. Peptide translation of such transcripts uses regular tRNAs but produces peptides identical to those resulting from decoding by tRNAs with expanded anticodons of regular transcripts [102]. These delRNAs are produced by systematic deletions, every third nucleotide, and correspond at deletion level, to systematic nucleotide substitutions/exchanges. This predicts that chimeric peptides consisting in part of regular-translated, and in part tetra- or pentacoded peptides, might exist.

The strongest evidence for swinger-encoding is the association between detected swinger RNAs and detected swinger peptides. Analyses detecting mass spectra matching predictions according to translations of tetra- and pentacodons suffer the caveat that evidence is based solely on mass spectra, with the above discussed difficulties in asserting the robustness of results based only on proteomics. However, further analyses detected peptides matching translations, according to expanded codons, of swinger-transformed sequences, and showed their association with detected swinger RNA [103]. Hence from a methodological point of view, translation according to expanded codons of swinger RNAs is stronger evidence for tetra- and pentacoding than such translation of regular RNA because it is confirmed by the independent detections of two ‘unusual’ types of molecules, swinger RNA and corresponding peptides matching expanded codons.

### 4.9 Robustness of experimental design

An anonymous reviewer indicated that analyzes comparing transcriptome and proteome make sense only if data originate from individuals with the same phenotypes, and if possible the same tissues and even the same individual(s), however analyzes compare tumor transcriptome [30] with normal proteome [33]. This setup is indeed suboptimal. However, considering this point, RNA and peptide data converge (also in previous analyzes, [102], [103]) despite that RNA and peptide data originate from different tissues/individuals/phenotypes. This indicates that the phenomenon is general, and robust. This should not be surprising, because analyzes consider only RNA and peptides corresponding to the mitogenome. Most tissue-specific differences in mitochondrial RNA and protein profiles relate to molecules imported from the cytosol [13].

Methods used to detect the various types of unusual peptides take into account the large numbers of possibilities in matching observed and hypothetical mass spectra, so that positive detections are robust, and could not be due to chance.

Beyond methodological issues, occurrence of peptides coded by combinations of presumably unusual coding systems (translation of stops, together with translation according to expanded codons, and this for swinger RNAs), suggests that these basically ignored mechanisms expand more frequently than presumed the coding potential of genes, at least of the short mitogenomes. Detections of chimeric peptides, consisting of peptide parts corresponding to regular translation, adjacent to peptide parts matching translation of contiguous swinger RNA, strengthen confidence in the validity of results as positive controls, and expand our understanding of the phenomenon: swinger peptides are occasionally integrated in regular mitochondrion-encoded proteins, but their occurrence is downregulated.

## 5 Conclusions

1. Analyses of MS/MS mass spectrometry data detect peptides matching the translation of chimeric transcripts, RNA following in part regular, and in part swinger-transformed transcription, assuming abrupt switches between regular and swinger transformed parts of the RNA.
2. The 186 detected chimeric peptides (peptides consisting of a part encoded by regular RNA and a contiguous part encoded by swinger RNA) represent 3/100,000 among potential chimeric peptides, about 200 times fewer (6/1000) than detected swinger peptides (peptides entirely encoded by swinger RNA) in the same data. Eleven among these 186 chimeric peptides have a regular-encoded part that corresponds to proteins translated from classical mitochondrion-encoded genes.
3. Chimeric peptides map on previously detected swinger RNA. This association is weaker than a previously described association between ‘regular’ swinger peptides and swinger RNAs [103].
4. The vertebrate mitochondrial genetic code differs from the nuclear genetic code for four codons. Numbers of detected chimeric peptides that could be translated from human mitogenome sequences according to the nuclear genetic code are significantly fewer than expected considering the differences between the two genetic codes. This means that the majority of detected chimeric peptides are not cytosolic contaminations and were translated in the mitochondrion.
5. Previous detections of swinger peptides (predicted products of translation of swinger RNA) suggested that swinger transformed RNA is translation-competent [103]. Chimeric peptides where the regular part corresponds to known mitochondrion-encoded proteins might be incorporated into the respiratory chain complexes. Chimeric and swinger peptides might affect known mitochondrial functions despite low abundances if they have regulatory functions. Results are compatible with the possibility that some proteins are encoded by swinger transformations, with yet unknown functions.

## Figures and Tables

**Table 1:** Human mitochondrial peptides detected assuming abrupt switches between regular and swinger parts of RNA, for peptides where each regular and swinger parts have > 8 amino acids (mass spectra from [33]). Columns are: 1. Peptide number; 2. swinger type; 3. amino acid inserted at stop(s) (‘no’ indicates lack of stops); 4. strand and frame; 5. peptide sequence; 6. PSMs; 7. Xcorr; 8. trypsin miscleavage; 9. PEP; 10–13. Positive strand positions of 5’ and 3’ extreme amino acids of regular and swinger parts of detected peptide; 14. Peptide extremity matching regular transcription. Underlined: peptide swinger part; *, $ marks swinger peptide parts covering previously described swinger reads, respectively previously described swinger peptides [103]. Peptides 8 and 9 differ in posttranslational amino acid modifications (not indicated). Highlighted peptide parts match both translations according to vertebrate mitochondrial, and nuclear (standard) genetic codes. Peptide parts not highlighted match only translation according to the vertebrate mitochondrial genetic code, and are incompatible with translation according to the nuclear genetic code. For example, peptide 3 could be translated in the cytosol on the base of RNA transcribed from mitochondrial inserts in the nuclear chromosome (numts), peptides 5 could not, as peptides 1 and 2 because at least one part of the peptide is not compatible with translation according to the nuclear genetic code. Further analyses (see text) show that fewer detected peptides are compatible with the nuclear genetic code than expected by chance, and that more peptides than expected by chance are compatible only with translation according to the mitochondrial genetic code.

1	2	3	4	5	6	7	8	9	10	11	12	13	14		
1	ac	a	r0	YGVSEGLAAPVGAYNVGAFAALYMAANFSFLNQDAVQVADK	3	2.77	0	0.374	3246	3156	3153	3123	5′	29	12
2	ac	v	r1	SCLLAFLMGSLMLTLIVGLSK	20	3.04	0	0.169	3465	3432	3492	3468	3′	12	9
3	ac	r	r0	AWGGGFDVDWWGSDDIVAMR	43	3.06	0	0.745	16188	16161	16158	16131	5′	10	10
4	ag	k	r2	AIGKVAFSTSVMLEVMFLVNK	185	3.06	1	0.082	2289	2262	2319	2292	3′	11	10
5	ag	f	r1	SYTFPPGSSSVACWLGCSPSPTLTLIFGLSK	49	3.38	0	1.000	3456	3432	3522	3459	3′	9	22
6	ag	s	r0	GGSPSDSTTSSQQLLSSILWSK	149	3.98	0	0.755	10125	10101	10164	10128	3′	10	12
7	ag	no	f0	LLGAVPLASASLTIGSLALAGMPFLTGFYSKDHIIETANMS	1	2.37	1	0.931	13704	13767	13644	13701	3′	29	12
8	at	no	f1	FIAYHSPGKVNFVPATAVTR	399	2.77	1	0.196	882	924	855	879	3′	11	9
9	at	no	f1	FIAYHSPGKVNFVPATAVTR	604	2.77	1	0.633	882	924	855	879	3′	11	9
10	at	p	f1	MPNSFNWDVGGNSSKLPVECLVEQGPEAR	1	2.31	1	0.605	1467	1500	1407	1464	3′	17	12
11	at	y	r2	TMSYALTLLLLQTCRGFSR	25	4.34	1	0.836	5601	5574	5631	5604	3′	9	10
12	at	no	r2	DMGDASVMGLSVNEASYDGK	19	2.17	0	0.121	6933	6906	6963	6936	3′	10	10
13	cg	no	f0	TLGQGVAHDLRTNPVDFVGDK	6	2.69	1	0.094	1344	1365	1368	1404	5′	9	12
14	cg	k	r2	LLASLPQPTVVPSTMPTISVRSGVLAGCLIGWWKPK	2	2.00	1	0.592	11103	11058	11163	11106	3′	16	20
15	cg	t	r0	TDNTNHHLTGSAIMTMTAPVK	540	4.89	0	0.653	11625	11601	11661	11628	3′	11	10
16	cg	no	r1	TSQTDLLTDPPITYEFLWAFSVNK$	8	2.03	0	0.592	12150	12126	12195	12153	3′	9	14
17	ct	m	f1	MWEDLMVEAMNSLSSATVGR	880	4.49	0	0.837	1995	2019	2022	2052	5′	9	11
18	ct	e	r0	GMGPMAYLASLALKENMVNNAEGFK	642	2.47	1	0.182	4233	4209	4206	4161	5′	9	16
19	ct	t	f0	NPSLSISVPSTRHVSMPITISSIPPQTTEMCLMK	3	2.54	1	0.866	4290	4350	4251	4287	3′	21	13
20	ct	a	r2	GVNWAKMNIAGYESSYNEQR	5	2.76	1	0.352	10512	10485	10542	10515	3′	10	10
21	ct	m	r0	AMMGDCAVCGTEMMSMCIK	13	2.03	0	0.42	13914	13890	13887	13860	5′	10	9
22	ct	v	r0	NVVWSVAVAAMMKGGVGVGMGGHMEMK	31	3.31	1	0.393	15321	15279	15276	15240	5′	14	13
23	ct	y	f1	DVSGPSSPSSSLMTLTLFSPDLLGDPDNYTLANPLNTPPY	2	2.27	0	0.724	15714	15801	15684	15711	3′	30	10
24	gt	q	r2	NNLFSLYCYLFQLWMMDPEHMNSMALK	1	2.55	0	0.464	13329	13287	13365	13332	3′	16	11
25	ac gt	s	f0	MVGSFMGSGDKPTEPGDSFGEPRSEAGPGPGSTLQSAR	38	2.43	1	0.459	1995	2049	2052	2109	5′	19	19
26	ac gt	h	r2	WSSSLAAPSAFVLVGMSSRHSLLVCGTHVYFFGHNWNK	1	2.71	1	0.218	4428	4389	4500	4431	3′	14	24
27	ac gt	s	f2	SGWVEWSRHSVLLLLSLPVLAAGITMLLTDR	8	3.85	1	1.000	6591	6648	6558	6588	3′	21	10
28	ac gt	y	f0	EATASSAGNDASYDGQSGKDSQATPYTKPTPK	9	2.77	1	0.425	7221	7254	7161	7218	3′	12	20
29	ac gt	x	r1	GDKLFYDXGLLWGAQAGMVR	435	4.06	1	0.901	7503	7479	7476	7446	5′	9	11
30	ac gt	x	r1	RPLSPXGASLWSSVLXTYLR	129	5.31	0	0.516	7476	7452	7509	7479	3′	9	11
31	ac gt	n	f0	VMVTDLLQKSWSPHSYNNYITNR	6	2.39	1	0.971	8157	8193	8127	8154	3′	13	10
32	ac gt	n	f2	SNALNNAGKNAEGHYSSSPNNK	1	2.52	1	1.000	8559	8583	8520	8556	3′	9	13
33	ac gt	c	f1	TPGVVPEPAPAANVHSSCPPCPWLPCFPPSLPPSLTLTK	15	2.75	0	0.296	12564	12624	12510	12561	3′	22	17
34	ac gt	no	f0	SLKQNWDFSFNSSTMVVAGIFLLIR	1	2.11	1	1.000	13299	13338	13266	13296	3′	14	11
35	ac gt	x	f1	EMHLCSXEDSRAHNTWGXLK	13	2.02	1	0.989	16728	16752	16698	16725	3′	10	10
36	ag ct	k	r2	SLAPSGWSLLNLTNPLFSSMNLPTILLHKR	16	4.31	1	0.208	1728	1704	1788	1731	3′	11	19
37	ag ct	d	r1	LGDDWLEDMGNSNQNQLK	3	2.08	0	0.899	3426	3399	3396	3375	5′	9	9
38	ag ct	no	f2	WALFLSGTDSSSVSLAPLAATGSWGGLNQTQLR	7	2.96	0	0.143	5043	5076	4980	5040	3′	12	21
39	ag ct	y	f0	NPPYTWSDYMSIFCFVVCLGGLR	15	2.08	0	0.875	7485	7515	7518	7554	5′	10	13
40	ag ct	e	r1	YVGVEDESAVTNTSTNLTLPTIGQPSNGKK	2	2.14	1	0.472	7809	7779	7776	7719	5′	10	20
41	ag ct	q	r2	GDACWGPVPSQLGGQGQAGVVKGLQGLHQQGGPQNGGR	1	2.24	1	0.804	9456	9387	9384	9342	5′	23	15
42	ag ct	no	f1	AHVEAPIAGSMVLAVTSPGSNNR	37	3.74	0	1.000	11604	11643	11646	11670	5′	14	9
43	ag ct	v	r1	RSPLPGDQVDYVVVHGGMSVQFLWAFSVNK	34	3.08	1	1.000	12180	12126	12213	12183	3′	19	11
44	ag ct	f	f1	FNPFFGFVGPITKPTLNFNK	914	3.51	0	0.423	14874	14904	14847	14871	3′	11	9
45	ag ct	v	r2	HVHPEPSDEVAAYGANSIRCVGVGVVVMLVR	1	3.23	1	0.721	15015	14979	15069	15018	3′	13	18
46	at cg	no	f2	SSLRPYTKCVVFLASEEVK	4	2.41	1	1.000	3135	3129	3126	3096	5′	9	10
47	at cg	e	r2	QAEVFLSLQSSSQNHCFMQHISSGESASYVVPEK	215	3.23	0	0.224	3858	3822	3921	3861	3′	13	21
48	at cg	a	r0	FEDNKWDSFIDFYQTYFLGLAGNAGDCNGYGDMSYK	1	2.82	1	1.000	4074	4002	4107	4077	3′	24	12
49	at cg	e	f2	GISWPKLDEEGGGPFEAGEAPAGLK	59	3.82	1	0.169	5847	5874	5799	5844	3′	9	16
50	at cg	k	f1	SIAGVDVAMAVSGTKTLYLLHSNTHHNR	1	3.31	1	0.886	6201	6231	6147	6198	3′	10	18
51	at cg	r	f2	WLPWLGCSCGWCRLITSTPTYFPHYSR	1	2.49	1	0.941	8070	8097	8022	8067	3′	11	16
52	at cg	c	r2	CYLVGAFHCNLHNQENCK	27	3.92	0	0.690	8247	8223	8220	8196	5′	9	9
53	at cg	e	r1	AGEGLLEVWKASEPNSAVAK	8	3.17	1	0.171	9072	9042	9039	9012	5′	10	10
54	at cg	e	f1	EIFLSLLPQVGSGMGGESSR	11	3.57	0	0.249	9648	9669	9672	9705	5′	9	11
55	at cg	h	f2	GFLCIKLSCVGGCPHLLASSLYYFLTK$	8	2.08	1	0.935	11037	11070	10992	11034	3′	13	17
56	at cg	x	r0	DGGNXGSQGXGAMSSHVPMMKMNLNVVLK$	38	2.25	1	0.766	12321	12291	12288	12237	5′	12	17
57	at cg	r	f0	RPRLTSLPSLLNDINTILWSGGSAGSVNMGSVGEFVGR	1	2.94	1	0.124	15687	15735	15738	15798	5′	18	20
58	acg	n	r2	NTTTLSRTLNVGAVMNNVMVDVAGFNGSLVK	60	2.68	1	1.000	3315	3255	3345	3318	3′	21	10
59	acg	e	f0	SEHTPQLPTETTSSALSDRR	159	3.52	1	0.141	5085	5109	5112	5142	5′	10	10
60	acg	g	r0	WGGSTTNGGEPTGGSTLVGGEYKLQGDR	173	2.52	1	0.351	6483	6450	6531	6486	3′	12	16
61	acg	q	r2	QLVEQPKDTVEWQDMVEVGYNVVR	3	5.28	1	0.401	6891	6852	6924	6894	3′	13	11
62	acg	t	f2	TSKPHPTTTPPPSSSTPLQGLQCGGVRGVQAHQGAGHVQGR	73	3.03	1	0.876	13167	13215	13218	13287	5′	17	24
63	acg	t	f2	TSKPHPTTTPPPSSSTQISPITCGGVRGVQAHQGAGHVQGR	29	2.99	1	0.866	13167	13230	13233	13290	5′	22	19
64	acg	no	f2	GKQEPGLEQLCASSAALGEIPLPNNNPPLPK	17	2.36	1	0.236	13989	14022	13932	13986	3′	12	19
65	acg	e	f0	GLPQHQVHHKPHKPHYETHTQQK	2	2.05	0	1.000	14862	14901	14835	14859	3′	14	9
66	acg	e	f0	GLPQHQVHHQPHKPHYETHTQQK	2	2.05	0	1.000	14865	14901	14835	14862	3′	13	10
67	acg	a	f1	GGFSGPSQILIATILCSFMGK	4	2.24	0	0.319	16266	16305	16242	16263	3′	12	9
68	act	e	r2	GENAGELEGWTATLSCSSPEGPTTLGPLR	6	2.59	0	0.156	3228	3186	3273	3231	3′	14	15
69	act	t	r1	KPAGASPAFFPGGGTSTLKPVDTGATLLTMEGETVSPGSVVK$	2	2.04	0	1.000	5598	5511	5508	5472	5′	30	12
70	act	k	f2	NLPILLLTNVEPQPFPPTPK	27	4.28	0	0.948	11097	11118	11064	11094	3′	9	11
71	act	k	f2	NLPILLLTNVDPQPFPPTPK	197	4.97	0	1.000	11097	11118	11064	11094	3′	9	11
72	act	n	f2	WPLQCRQMSTTSSNFCHFNPNNNPPLPK	3	2.93	1	0.970	13998	14022	13941	13995	3′	9	19
73	agc	r	f2	DQPNPLRPCPAPLTDAMAIR	45	2.19	0	0.272	3945	3969	3972	4002	5′	10	10
74	agc	k	r1	NNSSPIPVPVSSMVMPAAKTGK	365	3.86	1	0.382	6369	6336	6396	6372	3′	13	9
75	agc	v	r2	FQLQSMPVLGSVGGVGGAMGVMR	125	4.64	0	0.958	8157	8115	8184	8160	3′	14	9
76	agc	no	r2	TSYLLLQQPLLWWLCWFKLCVFWK	5	2.01	1	0.163	10749	10716	10785	10752	3′	12	12
77	agc	q	r1	MQQAAFALQWMLLWGQDWQQQWMR	8	2.16	0	0.264	12432	12390	12459	12435	3′	15	9
78	agc	w	r2	MSSTCGNDISMSRISGLFSAWGWK	4	2.04	1	0.739	13098	13068	13137	13101	3′	11	13
79	agc	q	f0	LQQMMQQADVLVAGIFLLIR	13	3.77	0	0.353	13314	13338	13281	13311	3′	9	11
80	agc	v	f0	IVAFSTSSQLGLMVLEVPVGVK	260	4.16	0	0.296	13458	13488	13491	13515	5′	13	9
81	agc	d	r1	FLVDLGLGGVGAGFGSDEGLDDGLCGVWCDFMTVSLLMNK	2	2.80	0	0.029	13971	13887	13884	13854	5′	30	10
82	agt	a	f0	TDAQSGGASAYKAHENILLR	1	2.25	1	1.000	2343	2370	2313	2340	3′	10	10
83	agt	n	f2	LIYSTSITLLPMTGGNGEGMR	194	4.00	0	0.586	5442	5475	5478	5502	5′	12	9
84	agt	d	f2	LIYSTSITLLPMTGGDGEGMR	61	3.96	0	0.460	5442	5475	5478	5502	5′	11	10
85	agt	r	r2	NGVSSSGGVEEGGVEVAVCLLLCVEWWLVRVCLVLLVR	1	2.41	1	0.180	6423	6369	6366	6312	5′	20	18
86	agt	g	r2	AASCGPPSCLPEVGINGGGNGMISTAAGGPGIVGAMNEANG*	1	2.29	0	0.275	8493	8409	8526	8496	3′	30	11
87	agt	a	r2	AAEGNSYAEEFYGEADAGGGYAVEAATAWGGPPLAEAVPR	1	2.43	0	0.518	10143	10068	10065	10026	5′	25	15
88	agt	d	r0	NYSSAMGACQGGSDESNDDGSGVCVWFARGPVVAAPGAVDR	6	2.55	1	0.953	13566	13491	13488	13446	5′	26	15
89	agt	y	f2	RIRPVEVVGAIPHYFLLQYPHHR*	1	2.81	1	0.173	13713	13749	13680	13710	3′	12	11
90	atc	h	f1	HPLWNLHVEGGFSSNTCAVRPAVMGNVESYMHKHK	12	2.55	1	0.124	1413	1452	1455	1515	5′	15	20
91	atc	q	f1	GTLTVQQQHNMPPTFLGNAR	61	3.19	0	0.659	2616	2643	2646	2673	5′	10	10
92	atc	v	r2	GLVIVVVKALGSVGGVGGAMGVMR	429	4.41	1	0.299	8157	8115	8187	8160	3′	14	10
93	atc	v	r2	GLVIVVVKAVGSVGGVGGAMGVMR	1057	4.48	1	0.140	8160	8115	8187	8163	3′	14	10
94	atc	x	r1	MXLMLMIALVAXEXIQMVVLFSVMAGMLGVVGWCR$	1	2.16	0	0.818	13263	13233	13335	13266	3′	11	24
95	atc	k	r0	FNYAFLGWGDWLLLWNYHMGMKVK	17	3.81	1	1.000	14049	14022	14019	13980	5′	10	14
96	atc	q	r2	LWLCSKGGQWLQLGFVMNQFLMNDPK	35	2.60	1	0.630	15408	15381	15378	15333	5′	11	15
97	atg	n	f0	TLGQGVAHEVANNGLHLRPLFSSCTR	1	2.13	0	0.076	1344	1389	1392	1419	5′	17	9
98	atg	d	r1	EPMMHQVSMGKKPVSGGDPPDEDDVDGIK	14	2.57	1	0.129	5061	5007	5088	5064	3′	19	10
99	atg	no	f0	TMASSSPPSVPPAPAGSASASVTVASPPLALVAPVR$	13	2.42	0	1.000	5376	5409	5412	5481	5′	9	27
100	atg	no	f2	GASFLFIWNSLYLLFGAWAGVLGTALSLLIRAELGQPGNL	1	2.24	1	1.000	6054	6141	6024	6051	3′	30	10
101	atg	m	r0	IMRMGAFGIGNMSGENTSTK	65	2.86	1	1.000	6291	6264	6261	6234	5′	10	10
102	atg	p	f0	APPTALVGTDSPHSTEAMWNDLLQCSEPPDSSFFSPPVA	10	2.64	0	0.564	6987	7074	6960	6984	3′	30	9
103	atg	k	r1	KPYTLPMESMNPFCSHSKAK	51	2.17	1	1.000	10284	10263	10314	10287	3′	10	10
104	atg	n	r1	GGAYQGNQSNNLLGGGLVVGWGLDNRLEGLFVVGLMSWSVG	3	3.02	1	0.924	12936	12846	12969	12939	3′	30	11
105	atg	x	r1	EWAEVSSCGEEGXGGADAASEEPTKTTGER	11	2.51	1	0.991	14826	14784	14781	14739	5′	10	19
106	cgt	q	r1	GLYWWDQQYGSGQGGWSLASPLDLGAQWTQGVGFR	1	2.15	0	1.000	228	189	186	126	5′	14	21
107	cgt	x	f0	HPKPKPWEMXLXIPLXIXLLVQXGTALWTLGK	1	2.72	0	0.632	2103	2166	2073	2100	3′	22	10
108	cgt	m	r0	LLQSDHTAFGSAPMSPQPK	2	2.13	0	0.217	2604	2577	2631	2607	3′	10	9
109	cgt	a	f2	AGASEGMTSYMMCLHTHYNLQHSPSNLANMSDK	6	2.06	0	0.034	4278	4347	4251	4275	3′	23	10
110	cgt	t	r2	RPPSGWPSCTSTLVSGATTTTGRGAGVTVETTECGSSTDNM	6	2.51	1	0.584	4989	4902	5028	4992	3′	30	11
111	cgt	no	r0	LFLGMCLKQENPVMMSGLK	393	4.09	1	0.162	12066	12033	12093	12069	3′	10	9
112	cgt	t	f0	VFLLTMTFNQNITLWIWQHIASTGHPGMNATMSCK	2	2.14	0	0.703	12330	12384	12387	12435	5′	19	17
113	cgt	f	f2	MNGTEGHVVHVPDGVASMIYPTLQLMFPTTNSPSK	8	2.57	0	0.942	13107	13161	13059	13104	3′	19	16
114	ctg	v	r0	VFVSLSLVSPFWLNPASTVAVNVNGYNEEVEVGHGYVVK	1	2.98	0	1.000	3126	3078	3192	3129	3′	17	22
115	ctg	e	f1	QSHMKSPEPVGDEEEDEER	26	4.14	1	0.032	3783	3807	3810	3837	5′	9	10
116	ctg	k	r2	DQVRPLVLCMVMLYFTIHLLHAYK	8	2.55	0	0.480	8601	8571	8568	8532	5′	12	12
117	ctg	k	f0	MNNKVFLVFVQTTIPLYLK	791	3.61	1	0.130	14001	14025	13971	13998	3′	9	10
118	ctg	no	f2	SPSSMYPNNKLEEGLYELK	70	4.56	1	0.048	15915	15945	15948	15972	5′	10	9
119	acgt	t	f1	GGAGGTPAATGTRTPSAGSDSVSTDFTQPTTSTTTPTNLK*	5	2.24	1	0.825	2520	2466	2586	2523	3′	18	22
120	acgt	a	r1	FVKAALFLLAGTYPSLGAR	79	4.05	1	0.342	2937	2913	2910	2883	5′	10	9
121	acgt	d	r1	MVEDMTGWADGLISTGDVDPTFSGVPKLSGGSAK	6	2.88	1	0.287	4305	4236	4233	4206	5′	23	11
122	acgt	f	r2	EEMLDGSFCGTFVFGGPVLALFSVMR	1	2.20	0	0.163	4452	4416	4413	4371	5′	14	12
123	acgt	no	r2	VPRQALVPFEVNEASYDGK	141	3.13	1	0.592	6930	6906	6960	6933	3′	9	10
124	acgt	no	r2	VPRQALVPFDVNEASYDGK	388	3.16	1	0.496	6930	6906	6960	6933	3′	9	10
125	acgt	no	f1	SNFLPTTLSRPIRNAPTLLGLGVMHAAHPAGQQMLEQAK	29	2.97	1	1.000	7287	7350	7353	7404	5′	21	18
126	acgt	q	r1	HGQAMLALPVLDPSVVVLGGCQGVGGK	1	2.74	0	0.031	10857	10833	10911	10860	3′	9	18
127	acgt	no	r2	VVGGVGWVPLAWLSLDMLQR	167	3.64	0	0.555	14448	14424	14481	14451	3′	9	11
128	acgt	a	r2	WMSGALILLGGAFCVLGSFRGGVGFVLLPGDVMADAGVER*	4	2.37	1	1.000	14772	14718	14715	14652	5′	18	22
129	actg	d	r1	SSYKDPFAEDADLDNDIALLSLGDLVPLVK	149	3.81	1	0.469	2733	2682	2769	2736	3′	17	13
130	actg	q	r2	GMGQGVHSQQAMVQAKVGAVMQQVMVDVAGGQNVGQPQGR	2	2.36	1	0.409	3363	3276	3273	3246	5′	30	10
131	actg	s	f0	NSVCSDGSARAVSPLAPGLSHK	25	2.75	1	0.560	3273	3303	3306	3315	5′	10	12
132	actg	s	f0	NSVCSDGSARSSSPLAPGLSHK	23	3.21	1	1.000	3273	3309	3312	3339	5′	12	10
133	actg	s	r0	MIMSAWSWKVMSSSMMETSMVEHLLDMIEIRPR	6	2.41	1	0.948	7527	7482	7479	7431	5′	17	16
134	actg	no	f2	SLSPFMITPSSVGVGVMVAVER	59	3.28	0	0.884	7767	7794	7797	7830	5′	11	11
135	actg	t	r0	TPKEQEIGEATVGTGIFNLTAK	2	2.42	1	1.000	8778	8748	8811	8781	3′	11	11
136	actg	no	f2	NQMIQALLITILLGLYFTLLSIVTAGTVFGLR	1	2.29	0	0.690	9834	9894	9897	9930	5′	20	12
137	actg	f	r1	SNAFGESKFTPTETMADTMAAFFFEYCGK	1	2.42	1	0.403	11400	11367	11451	11403	3′	12	17
138	actg	r	r2	WGSSNHEHGGAGCLMGMVQGR	571	4.35	0	0.165	11481	11460	11517	11484	3′	9	12
139	actg	k	r2	WGSSNHEHGGAGGLMGMVQGKGK	67	4.67	1	0.973	11478	11454	11517	11481	3′	10	13
140	actg	d	r0	AMLLDMGAWVSKVETWVDAR	9	4.42	1	1.000	12186	12153	12150	12126	5′	11	9
141	actg	d	r0	AMLLDMGAWVSQVETWVDAR	9	4.42	0	1.000	12186	12153	12150	12123	5′	10	10
142	actg	no	f2	VSAASSGFYRPLPNNNPPLPK	81	3.67	0	1.000	13992	14022	13962	13989	3′	11	10
143	agct	k	r2	SKTLLLMWTLILIQSTSGK	110	3.76	1	0.377	2757	2730	2727	2703	5′	10	9
144	agct	s	f0	ASLSVALDPSGSTNTSLTAKHPNQLASIYFSR	2	2.24	1	0.987	5775	5832	5730	5772	3′	20	12
145	agct	no	f0	KAPNPCLAICALDACMLEK	1222	3.24	1	0.448	5931	5955	5958	5985	5′	9	10
146	agct	s	f0	GGPAGSVFWATIQANPMASMTFSKSYSK	4	2.67	1	0.429	7608	7653	7572	7605	3′	16	12
147	agct	h	r0	TLLNKTSPTFTYLSGEAHLTCHGEK*	4	2.19	1	0.342	13599	13569	13641	13602	3′	11	14
148	agtc	h	f0	SLLPSLSTQHHRYSGWVPGGSGLDIHAPK	1	2.81	1	0.420	2445	2472	2475	2529	5′	12	17
149	agtc	r	f2	AATDDERPTTTGLNSSTTTLLLSR	5	2.66	0	0.303	5214	5247	5163	5211	3′	11	13
150	agtc	e	f1	LTEPLTNGESSWEKASGSMVLAAVLLK$	155	2.13	1	0.932	11628	11658	11580	11625	3′	11	16
151	agtc	m	f0	IVAFSTSSQLGLMASEALAGMK	168	3.53	0	0.790	13458	13494	13497	13521	5′	13	9
152	agtc	w	f0	IVAFSTSSQLGLMASEALAGWK	434	4.72	0	0.715	13458	13494	13497	13521	5′	13	9
153	agtc	d	f0	IVAFSTSSQLGLMASEALAGDK	448	4.33	0	0.817	13458	13494	13497	13521	5′	12	10
154	agtc	r	r1	SLLPFVLFTFTPWSTLGLSMFLWVLGRGGLLFGR*	1	3.40	1	1.000	13758	13725	13821	13761	3′	13	21
155	agtc	no	r2	VAPVMSLIWFVLLPVSGPILEWCGRLMK	16	3.06	1	0.437	14934	14907	14991	14937	3′	9	19
156	atcg	k	r2	ATKTVGGVFGQTNQSPDPK*	1	2.68	1	0.251	321	294	291	267	5′	10	9
157	atcg	y	f1	SVGGGSAGYCVCGAAWGLGEPTKPQYHPPQFMYLTSSK	8	2.37	0	0.517	555	609	498	552	3′	19	19
158	atcg	e	r0	VISSEFIMQSQSPKHELEK	42	3.08	1	0.103	1629	1605	1602	1575	5′	10	9
159	atcg	a	f2	LYSQAFNSSSAQHTHGVGGCGVHWVRFEFK	1	2.25	1	0.669	3324	3369	3372	3411	5′	15	25
160	atcg	no	f2	LMPPLCKIHHESVALLVR	20	2.55	1	1	3936	3912	3909	3885	5′	9	9
161	atcg	g	r1	EKNQAVPEGPSMFISGPTQVK	3	2.47	1	0.573	4383	4353	4413	4386	3′	11	10
162	atcg	g	r1	EKNQAVPEGLAMFISGPTQVK	15	2.81	1	0.57	4380	4353	4413	4383	3′	11	10
163	atcg	g	r1	EKNQAVPEGLSMFISGPTQVK	20	2.80	1	0.57	4383	4353	4413	4386	3′	11	10
164	atcg	no	f0	AHTPKMLVMGPGLLPSGQGLGR	27	2.13	1	0.472	4503	4527	4530	4566	5′	10	12
165	atcg	x	r1	SEASASGSAKAAHDHLDDHPMMXLLFFVNSSMMAHLGK	1	2.79	1	0.34	5115	5064	5175	5118	3′	18	20
166	atcg	q	r2	QDCCDQDGSDEDPSNQNPQPAPKQER	1	2.42	1	0.309	6315	6282	6279	6240	5′	12	14
167	atcg	v	r2	GPVTVQAKVVGSVGGVGGAMGVMR	203	4.83	1	0.074	8160	8115	8187	8163	3′	15	9
168	atcg	v	r2	GPVTVQAKVLGSVGGVGGAMGVMR	181	4.59	1	0.082	8157	8115	8187	8160	3′	14	10
169	atcg	a	f2	AASHPVPVPMTLLMLGLLTNTLTMYQWWR	24	2.66	0	0.053	9477	9537	9450	9474	3′	19	10
170	atcg	a	r0	AMTLHAHAGAMFSEPAVLWVAISAMSAGAEPTAVANAK	2	2.44	0	0.693	11196	11115	11226	11199	3′	28	10
171	atcg	q	r2	GDAGEMLLVNAGLLGAQFLLASK	28	3.47	0	0.068	13719	13695	13692	13653	5′	10	13
172	atcg	e	r2	GDAGEMLLVIAGLLGAEFLLASK	29	3.54	0	0.147	13719	13695	13692	13653	5′	10	13
173	atcg	t	r0	SAEHSLGAGYHSGLMWGGVFKGLATVTLSGSPTTSGENT	1	2.93	1	0.885	15546	15456	15573	15549	3′	30	9
174	atgc	c	f2	NIPFLLFGVNSCCVIPSCNMPSACWINCKCLCK	1	2.36	1	0.239	1872	1911	1815	1869	3′	13	19
175	atgc	e	f0	SVESMLLGEENNFAEEAKAK	641	3.52	1	0.148	1902	1929	1872	1899	3′	11	9
176	atgc	w	f0	WDISQGKTFAVILNLVLYPHPPK	23	3.62	1	0.851	3240	3270	3204	3237	3′	11	12
177	atgc	k	f1	HYLYDMSPLNGIENHGKK	154	3.19	1	0.419	4272	4293	4296	4323	5′	9	9
178	atgc	h	f1	LMHHHYKSSAHHVHSPMIVHHNNYQYK*	35	2.40	1	0.591	6501	6522	6444	6498	3′	9	18
179	atgc	e	r1	IINITAVEENPSGRSSLHK	32	3.62	1	0.040	7155	7131	7128	7101	5′	10	9
180	atgc	e	r1	IINITAVEEIPSGRSSLHK	42	4.38	1	0.156	7155	7131	7128	7101	5′	10	9
181	atgc	no	f2	LLKECLSLASVPATPPYHTFEEPVYMK	12	2.48	1	0.681	7524	7560	7482	7521	3′	13	14
182	atgc	f	f1	AQWLFAFALFLKMPFPFEVMFHMSMK	5	2.10	1	1.000	9006	9051	9054	9081	5′	16	10
183	atgc	n	r1	NYLYYKSYCVSYSTTNNLSFNITK	14	3.54	1	0.304	11298	11268	11265	11229	5′	12	12
184	atgc	k	r0	SKNKPDTNASSNPVMMSGLK	232	3.49	1	0.793	12054	12033	12087	12057	3′	9	11
185	atgc	c	f1	VIFCQMVEFCVMVQVHSDNCADIIEAPLHKMTSK	1	2.13	1	0.623	13428	13452	13353	13425	3′	9	24
186	atgc	y	f1	VVYNGLQAMPEAYSQDFSLLTTFPPHPPSK	12	2.47	0	0.484	13941	14001	13914	13938	3′	21	9

**Table 2:** Frequencies and lengths of chimeric swinger peptides detected in Table 1: Columns indicate: swinger type; peptide number; mean PSMs; mean amino acid number in peptide non-swinger part; mean amino acid number in peptide swinger part.

Swinger type	N	PSMs	Reg	Swinger
A ↔ C	3	22.0	17.0	10.3
A ↔ G	4	96.0	14.8	14.0
A ↔ T	5	209.6	11.6	10.0
C ↔ G	4	139.0	11.3	14.0
C ↔ T	7	225.1	14.7	11.7
G ↔ T	1	1.0	16.0	11.0
A ↔ C + G ↔ T	11	59.6	13.8	14.2
A ↔ G + C ↔ T	10	103.0	13.2	14.4
A ↔ T + C ↔ G	12	31.2	12.3	14.8
A → C → G → A	10	52.2	14.6	13.7
A → C → T → A	5	47.0	14.2	13.6
A → G → C → A	9	91. 9	14.1	10.2
A → G → T → A	8	33.3	18.3	12.4
A → T → C → A	7	230.3	12.1	14.7
A → T → G → A	9	18. 8	18.3	12. 8
C → G → T → C	8	52.4	18.4	12.9
C → T → G → C	5	179.2	11.4	12.6
A → C → G → T → A	10	82.1	14.0	14.3
A → C → T → G → A	14	71.8	13.6	11.9
A → G → C → T → A	5	268.4	13.2	11.4
A → G → T → C → A	7	173.1	12.1	13.6
A → T → C → G → A	18	33.7	14.6	12.3
A → T → G → C → A	13	92.6	11.8	12.7

**Table 3:** Chimeric peptides from Table 1 with regular part matching proteins translated from known mitogenome-encoded genes. Swinger parts are underlined, gene identity is followed by the position of the ‘normal’ part of the peptide matching the regular translation of the gene in the regular protein. The swinger transformation and the amino acid inserted at stop(s) are also indicated. Peptide parts matching translation according to both nuclear and mitochondrial genetic codes are highlighted: peptide 100 could be translated in the cytosol on the base of RNA transcribed from mitochondrial inserts in the nuclear chromosome (numts), all remaining peptides could not, as at least on part of the peptide is incompatible with translation according to the nuclear genetic code. Analyses (see text) show that there are fewer detected peptides compatible with the nuclear genetic code than expected, and more than expected peptides compatible only with the mitochondrial genetic code.

Table 1 #	Peptide	Gene	Position	Swinger rule	Stop
100	GASFLFIWNSLYLLFGAWAGVLGTALSLLIRAELGQPGNL	COX1	18–47	A > T > G	r
27	SGWVEWSRHSVLLLLSLPVLAAGITMLLTDR	COX1	205–213	A ↔ C + G ↔ T	s
181	LLKECLSLASVPATPPYHTFEEPVYMK	COX1	500–512	A > T > G > C	x
136	NQMIQALLITILLGLYFTLLSIVTAGTVFGLR	COX3	157–168	A > C > T > G	e
169	AASHPVPVPMTLLMLGLLTNTLTMYQWWR	COX3	41–59	A > T > C > G	a
23	DVSGPSSPSSSLMTLTLFSPDLLGDPDNYTLANPLNTPPY	Cyt B	238–267	C ↔ T	y
19	NPSLSISVPSTRHVSMPITISSIPPQTTEMCLMK	ND1	305–318	C ↔ T	t
38	WALFLSGTDSSSVSLAPLAATGSWGGLNQTQLR	ND2	165–176	A ↔ G + C ↔ T	n
34	SLKQNWDFSFNSSTMVVAGIFLLIR	ND5	249–262	A ↔ C + G ↔ T	f
80	IVAFSTSSQLGLMVLEVPVGVK	ND5	301–313	A > G > T > C	d
7	LLGAVPLASASLTIGSLALAGMPFLTGFYSKDHIIETANMS	ND5	374–402	A ↔ G	
